# Distinct Defects in Spine Formation or Pruning in Two Gene Duplication Mouse Models of Autism

**DOI:** 10.1007/s12264-017-0111-8

**Published:** 2017-03-03

**Authors:** Miao Wang, Huiping Li, Toru Takumi, Zilong Qiu, Xiu Xu, Xiang Yu, Wen-Jie Bian

**Affiliations:** 1 Institute of Neuroscience, State Key Laboratory of Neuroscience, CAS Center for Excellence in Brain Science and Intelligence Technology, Chinese Academy of Sciences, Shanghai, 200031 China; 2 University of Chinese Academy of Sciences, Beijing, 100049 China; 3 Department of Child Healthcare, Children’s Hospital of Fudan University, Shanghai, 201102 China; 4 RIKEN Brain Science Institute, Wako, Saitama, 351-0198 Japan; 5 School of Life Science and Technology, ShanghaiTech University, Shanghai, 201210 China

**Keywords:** Autism, Autism spectrum disorder, Spine, Spine formation, Spinogenesis, Spine pruning, Gene duplication, MECP2, 15q11-13 duplication

## Abstract

Autism spectrum disorder (ASD) encompasses a complex set of developmental neurological disorders, characterized by deficits in social communication and excessive repetitive behaviors. In recent years, ASD is increasingly being considered as a disease of the synapse. One main type of genetic aberration leading to ASD is gene duplication, and several mouse models have been generated mimicking these mutations. Here, we studied the effects of *MECP2* duplication and human chromosome 15q11-13 duplication on synaptic development and neural circuit wiring in the mouse sensory cortices. We showed that mice carrying *MECP2* duplication had specific defects in spine pruning, while the 15q11-13 duplication mouse model had impaired spine formation. Our results demonstrate that spine pathology varies significantly between autism models and that distinct aspects of neural circuit development may be targeted in different ASD mutations. Our results further underscore the importance of gene dosage in normal development and function of the brain.

## Introduction

Autism spectrum disorder (ASD) encompasses a wide range of neurological disorders with developmental origins, displaying a variety of symptoms and all including the key characteristics of impaired social interaction and excessive repetitive behaviors and/or restricted interests [1]. These behavioral abnormalities are thought to be caused by alterations in neural circuits. Based on accumulating evidence demonstrating defects in synaptic development and/or function in animal models of ASD and in patients, autism has been considered as the disease of synapse [2–7]. In fact, many genes underlying ASD encode molecules that directly participate in and/or regulate synaptic structure and function, including pre- and postsynaptic scaffolds, subunits of neurotransmitter receptors and synaptic adhesion molecules [4–6, 8–10]. Over 90% of excitatory synapses in the brain are located on dendritic spines [11], which are small and thorn-like protrusions extending from the dendritic shaft. Spines undergo dramatic changes during development, in species ranging from rodents to humans [12–19].

Spines form rapidly during early postnatal life *via* a process called “spinogenesis”, which usually lasts 3–4 postnatal weeks in mice [14, 17]. During adolescence, the brain undergoes a course of spine “pruning” or elimination, to remove excessive synaptic connections and strengthen the physiologically useful/relevant connections [12–16, 18–21]. Both spinogenesis and spine pruning contribute significantly to efficient neural circuit wiring and normal brain function. In addition to their number, the size and shape of spines are also important [22]. It has been shown that the volume of spine head tightly correlates to the area of postsynaptic density, as well as presynaptic vesicle number [23], while the spine neck tunes the postsynaptic response and also shows plasticity upon changes in presynaptic inputs [24, 25]. Thus, measuring the number and morphology of spines is potentially a simple and effective way to assess changes in the synaptic connectivity in ASD.

Up to date, hundreds of genes and genetic alterations have been linked to ASD, including those affecting synaptic function, those regulating gene transcription and post-transcriptional modification, and those involved in other important biological processes [2, 4–6, 8–10, 26–29].

It is of particular interest that copy number variations (CNVs), large nucleotide (one kilobase to a few megabases) duplications or deletions, contribute significantly to ASD. CNVs can occur by inheritance or *de novo* mutation. *De novo* CNV occurs in an offspring whose parents do not have the genetic change and is more common in ASD than inherited cases [9, 26–30]. Of the reported CNVs in ASD, several are highly interesting in that both their deletion and duplication lead to autistic phenotypes. A well-characterized example is the X-linked *Methyl-CpG-binding protein 2* (*MECP2*), loss-of-function of which results in Rett syndrome [31–33] while its duplication leads to many autistic symptoms including lack of eye contact and verbal communication, loss of speech, restricted interests and stereotypic behaviors [33–35]. Another example is the 15q11-13 region of human chromosome 15, which includes a series of imprinting genes, as well as non-imprinting ones. Maternal deletion of this region results in Angelman syndrome, paternal deletion leads to Prader-Willi syndrome, while its duplication represents one of the most frequently reported CNVs in ASD. All the three mutations share ASD features [27, 30, 36]. The observation that deletion and duplication of the same gene or chromosomal region can result in a similar phenotype underscores the importance of gene dosage to neural circuit development and function [5, 26, 27, 29, 30, 33].

In recent years, a number of mouse models of human ASD mutations have been generated. Here we examined two of the better-characterized gene/chromosomal duplication mouse models, the *MECP2* ^Tg1^ mouse model of *MeCP2* duplication syndrome [33, 37], and the 15q11-13 duplication mouse model that mimics duplication of human chromosome 15q11-13 region [36, 38]. We quantitated changes in spine density and morphology in these mice in the primary somatosensory and visual cortices at different developmental stages, as indicators of changes in neural circuitry.

## Materials and Methods

### Animals

All experimental procedures were approved by the Institutional Animal Care and Use Committee of the Institute of Neuroscience, Chinese Academy of Sciences (Shanghai, China), under protocol No. NA-003-2016. The hemizygote *MECP2* ^Tg1^ mice (full name: FVB-Tg (*MECP2*)1Hzo/J; JAX Stock No: 008679**)** [37] express full-length human *MECP2* under the endogenous human promoter, with hemizygote males expressing the protein at ~2-fold wildtype levels in the brain [37]. Only male *MECP2* ^Tg1^ mice and age-matched wild-type littermates (all on FVB background) of 1 and 3 months were used. The mouse model of human 15q11-13 duplication (on C57/BL6 background) carries an interstitial duplication of 6 Mb on mouse chromosome 7B-C that corresponds to human chromosome 15q11-q13, as previously described [38]. Both male and female mice and age-matched wild-type littermates at postnatal day 14 (P14) and 1 month were used, as the estrous cycle does not affect the spines in female mice at this developmental stage.

### Golgi Staining

Golgi staining was performed using the FD Rapid GolgiStain™ Kit (FD NeuroTechnologies, Columbia, MD), according to the manufacturer’s instructions. Briefly, mice were deeply anesthetized with 0.7% pentobarbital sodium. The freshly dissected brain was immersed into a mixture containing equal volumes of solution A and B at room temperature for approximately 10 days. The brains were then transferred into solution C for at least 48 h. Coronal sections (150 μm) were prepared with a freezing microtome. Sections were stained using solutions D and E after mounting onto the slides.

### Image Acquisition and Analysis

Stained sections were imaged using a Zeiss LSM PASCAL confocal microscope (Carl Zeiss, Jena, Germany), equipped with a 63× oil immersion Plan-Apochromat objective (N.A = 1.4) and at 2× optical zoom. The basal dendrites of layer 2/3 pyramidal neurons in S1BF and V1 were imaged. All images were coded with computer-generated random number sequence (https://www.random.org/sequences/) at the time of acquisition and analyzed blinded to the experimental condition. Original images were used to measure dendrite branch length and count spine number using Image-Pro Plus (Media-Cybernetics, Silver Spring, MD). Spine density was calculated as the number of spines per micrometer dendrite. Protrusions longer than 3 μm were considered as filopodia and were analyzed separately for P14 mice. The criterion for spine subtype classification was as previously described [15]. The proportion for each spine subtype was calculated as the percentage of total spines on the dendritic segment. Only images with sufficient quality to clearly distinguish and measure the spine shape were used for spine subtype classification. For example images, bright field original images were projected at minimal intensity and inverted, followed by background subtraction and brightness/contrast adjustment, using Fiji/ImageJ (NIH, Bethesda, MD). Paired example images were adjusted with the same parameters.

### Statistics

Statistical tests were carried out using GraphPad Prism 5 (GraphPad software, La Jolla, CA). Two-tailed Student’s *t*-test was used for comparison between pairs of samples, while one-way ANOVA followed by Tukey’s *post hoc* test was used for comparison between multiple samples. For spine subtype classification, two-way ANOVA followed by Bonferroni *post hoc* test was used. Data were collected from 3–6 mice for each condition, up to 10 images per mouse. Results are shown as mean ± SEM, and “*n*” refers to the number of dendrites or neurons. All conditions statistically different from control are indicated: n.s., not significant; *P < 0.05; **P < 0.01; ***P < 0.001.

## Results

### Impaired Spine Pruning and Maturation in the Primary Somatosensory Cortex of *MECP2*^Tg1^ Mice

*MECP2* is one of the earliest autism genes identified. Its loss-of-function mutations result in Rett syndrome [32, 33, 39], while its overexpression leads to progressive neurological symptoms with ASD features. Mice with doubled expression of *MECP2* (*MECP2* ^Tg1^) [37] show a series of progressive symptoms including social interaction deficits, aggressiveness, anxiety, behavioral seizures and abnormal electroencephalographic traces [37, 40], similar to those observed in *MECP2* duplication patients. These phenotypes can be rescued by re-normalizing *Mecp2* expression in mice [41]. More recently, transgenic monkeys overexpressing human *MECP2* were shown to recapitulate the key behavioral features of ASD, including impaired social interaction and stereotypic behavior [42]. Importantly, these autism-like behavioral defects were passed onto their offspring through germline transmission [42].

**Fig. 1:**
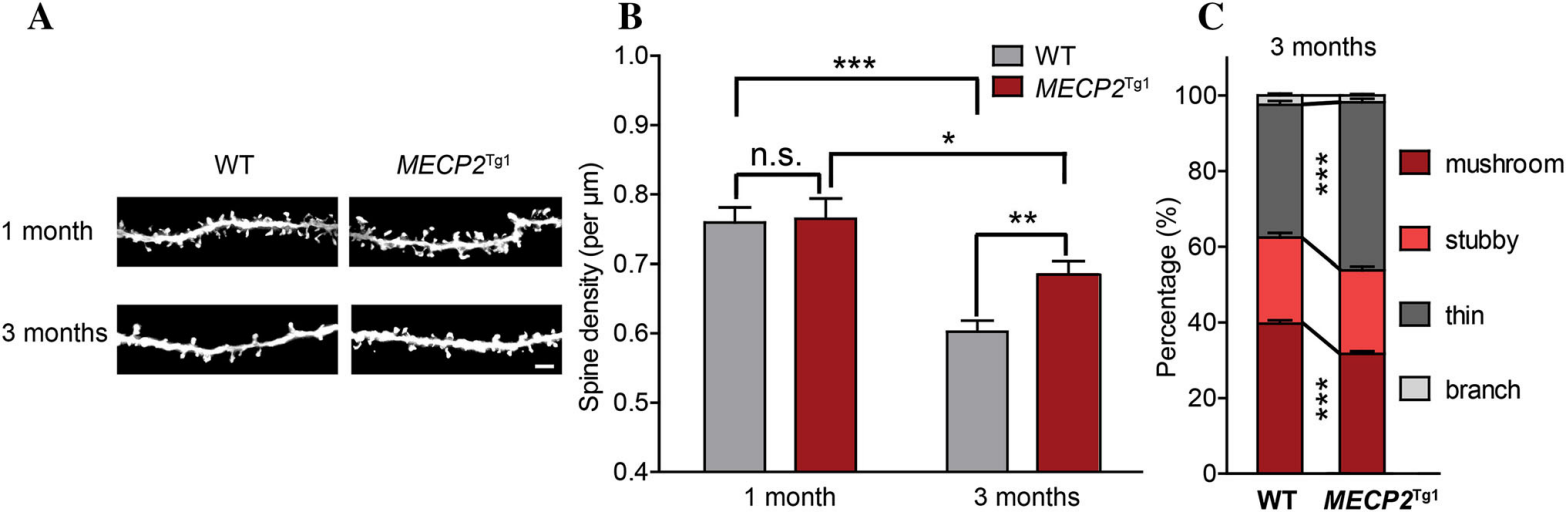
Spine pruning is impaired in *MECP2* ^Tg1^ mice in S1BF. **A** Representative inverted Golgi staining images showing spines in basal dendrites of layer 2/3 pyramidal neurons in S1BF, genotype and age as indicated. *Scale bar* 5 μm. **B** Spine density in wildtype (WT) and *MECP2* ^Tg1^ mice at 1 month (WT, *n* = 38; *MECP2* ^Tg1^, *n* = 25) and 3 months (WT, *n* = 44; *MECP2* ^Tg1^, *n* = 59). **C** Spine type classification for WT and *MECP2* ^Tg1^ mice at 3 months. Data are presented as mean ± SEM. *n.s.* not significant, *P < 0.05, **P < 0.01, ***P < 0.001.

Here we used the *MECP2* ^Tg1^ mice [37] to investigate the effect of *MECP2* overexpression on dendritic spine density and morphology in the primary sensory cortices at different developmental stages using Golgi staining. In previous work, we showed that spines in multiple sensory/motor cortices underwent activity-dependent pruning between 1 and 3 months of post-natal development [15]. Since spine pruning is highly development- and activity-dependent in the basal dendrites of layer 2/3 pyramidal neurons in the barrel field of primary somatosensory cortex (S1BF) [15], we first assayed these neurons in *MECP2* ^Tg1^ mice. The results showed that spine density was not significantly different between *MECP2* ^Tg1^ mice and wildtype littermate controls at 1 month (*P* > 0.05; Fig. 1A, B), indicative of normal spinogenesis. By the age of 3 months, spines in S1BF of wildtype mice have undergone spine pruning, as indicated by the substantial reduction in spine density (*P* < 0.001; Fig. 1A, B). However, in *MECP2* ^Tg1^ mice, spine density at 3 months was significantly higher than that of wildtype littermates (*P* < 0.01; Fig. 1A, B), and was only slightly lower than that of 1-month *MECP2* ^Tg1^ mice (*P* < 0.05; Fig. 1A, B; percentage reduction in spine density between 1 and 3 months: wildtype, 23.1%; *MECP2* ^Tg1^, 10.4%), suggesting that spine pruning was impaired in *MECP2* ^Tg1^ mice.

The pruning of some spines during the transition through adolescence is accompanied by and coordinated with the strengthening and maturation of the surviving ones [15]. To examine whether *MECP2* overexpression also affected spine maturation, we analyzed spine maturity in *MECP2* ^Tg1^ mice and wildtype littermates by sorting spines into 4 subtypes based on morphological criteria described previously [15]. Spines with mushroom-like shapes typically contain larger postsynaptic densities and a well-constricted spine neck, and thus are thought to be mature and stable, while thin spines are found to be more motile and immature [18, 21, 22]. We found that at 3 months, the percentage of thin spines significantly increased while that of mushroom spines decreased in *MECP2* ^Tg1^ mice, as compared to the wildtype littermates, suggesting that along with the spine pruning defect, more spines failed to mature in *MECP2* ^Tg1^ mice. Together, these results showed that both spine pruning and spine maturation were impaired in S1BF of 3-month *MECP2* ^Tg1^ mice, while spinogenesis at 1 month was essentially intact.

### Impaired Spine Pruning and Maturation in the Primary Visual Cortex of *MECP2*^Tg1^ Mice

**Fig. 2:**
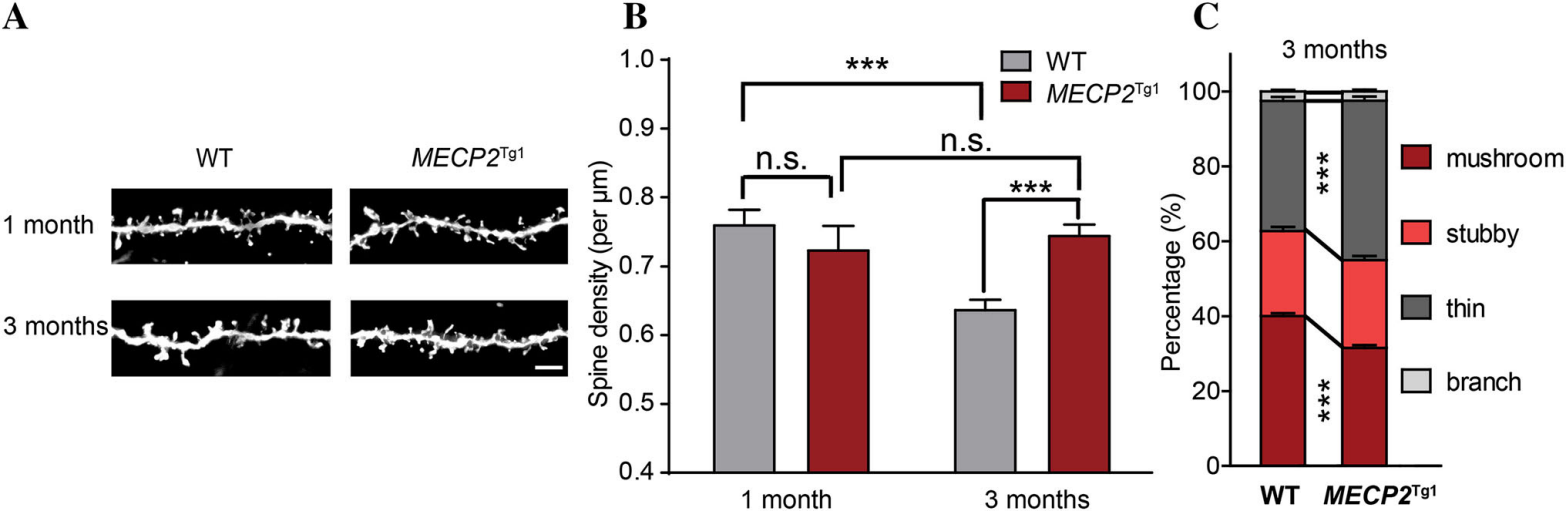
Spine pruning is impaired in *MECP2* ^Tg1^ mice in V1. **A** Representative inverted Golgi staining images showing spines in basal dendrites of layer 2/3 pyramidal neurons in V1, genotype and age as indicated. *Scale bar* 5 μm. **B** Spine density in WT and *MECP2* ^Tg1^ mice at 1 month (WT, *n* = 32; *MECP2* ^Tg1^, *n* = 26) and 3 months (WT, *n* = 44; *MECP2* ^Tg1^, *n* = 54). **C** Spine type classification for WT and *MECP2* ^Tg1^ mice at 3 months. ***P < 0.001.

To determine whether the defects of spine pruning and maturation observed in S1BF are specific to the somatosensory cortex or common to multiple sensory modalities, we further examined spine density and morphology in the primary visual cortex (V1). Similar to our results in S1BF, spine pruning, but not spinogenesis, showed significant defects in V1 of *MECP2* ^Tg1^ mice, as demonstrated by the significant differences in spine density between wildtype and *MECP2* ^Tg1^ mice at 3 months (*P* < 0.001) but not at 1 month (*P* > 0.05) (Fig. 2A, B). Notably, although spine density in S1BF of *MECP2* ^Tg1^ mice was slightly lowered at 3 months as compared with that at 1 month (*P* < 0.05; Fig. 1A, B), in V1 no reductions were observed (*P* > 0.05; Fig. 2A, B; wildtype: 1 month, 0.76 ± 0.02, 3 months, 0.64 ± 0.02; *MECP2* ^Tg1^: 1 month, 0.72 ± 0.04, 3 months, 0.74 ± 0.02). Consistently, an increased portion of thin spines and a decreased portion of mushroom spines were found in V1 of *MECP2* ^Tg1^ mice at 3 months (Fig. 2C). The overall pattern and extent of changes in S1BF and V1 were very similar, suggesting that *MECP2* duplication likely results in global defects in spine pruning and maturation in the sensory cortices.

### Spinogenesis is Impaired in 15q11-13 Paternal Duplication Mice

**Fig. 3:**
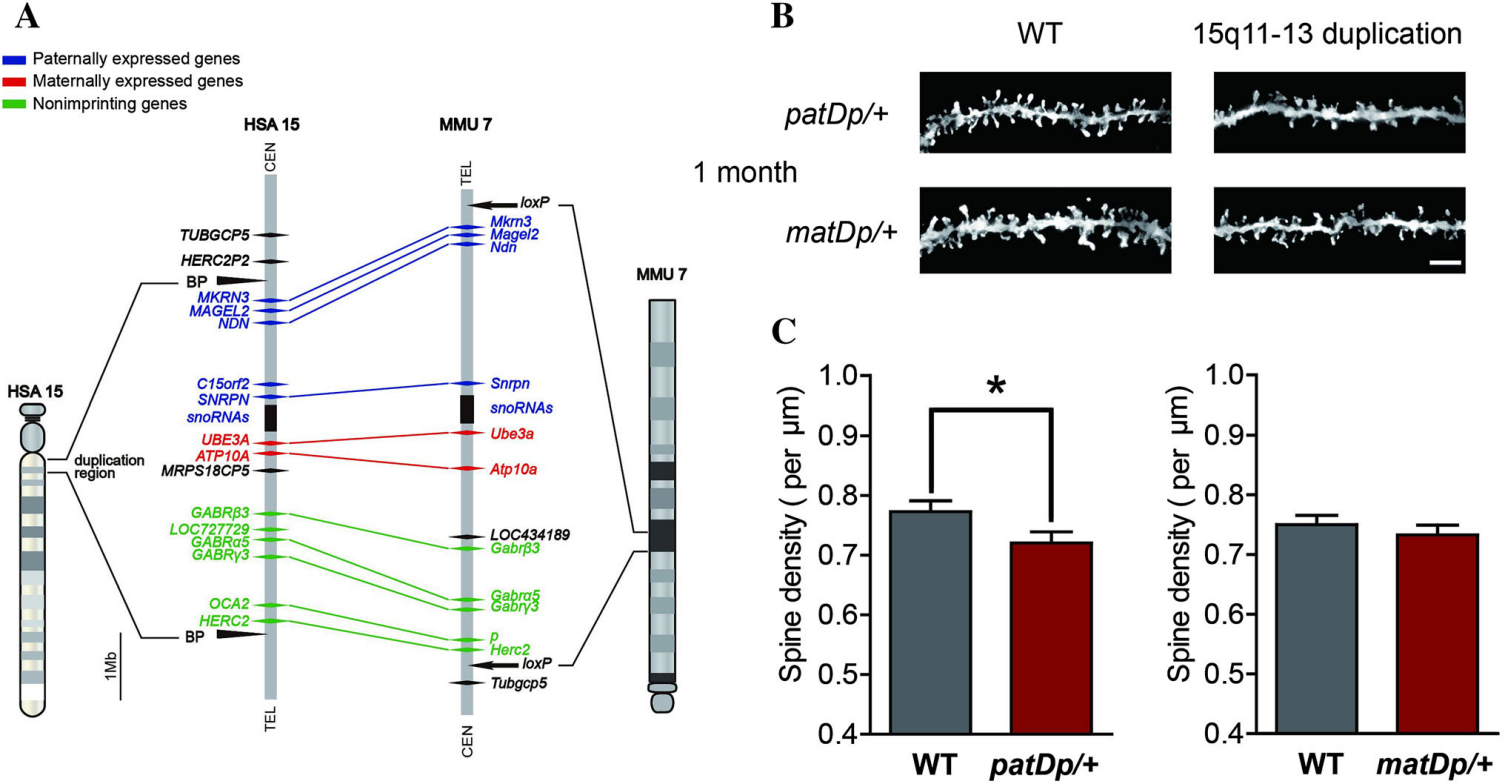
*patDp*/+ mice, but not *matDp*/+ mice, show delayed spine maturation at 1 month. **A** Schematic representation of the 15q11-13 duplication region in human chromosome 15 (*left*) and the corresponding region in mouse chromosome 7 (*right*). Genes expressed paternally, maternally, and nonimprinting genes are respectively marked in blue, red and green. Arrowheads indicate the border of duplication segments. Schematic took reference from previous literatures [38, 47]. **B** Representative inverted Golgi staining images showing spines in basal dendrites of layer 2/3 pyramidal neurons in S1BF of 1-month 15q11-13 duplication mice, genotypes as indicated. *Scale bar* 5 μm. **C** Spine density in WT (*n* = 34) and *patDp*/+ (*n* = 25) mice at 1 month. **D** Spine density in WT (*n* = 38) and *matDp*/+ (*n* = 37) mice at 1 month. *P < 0.05.

**Fig. 4:**
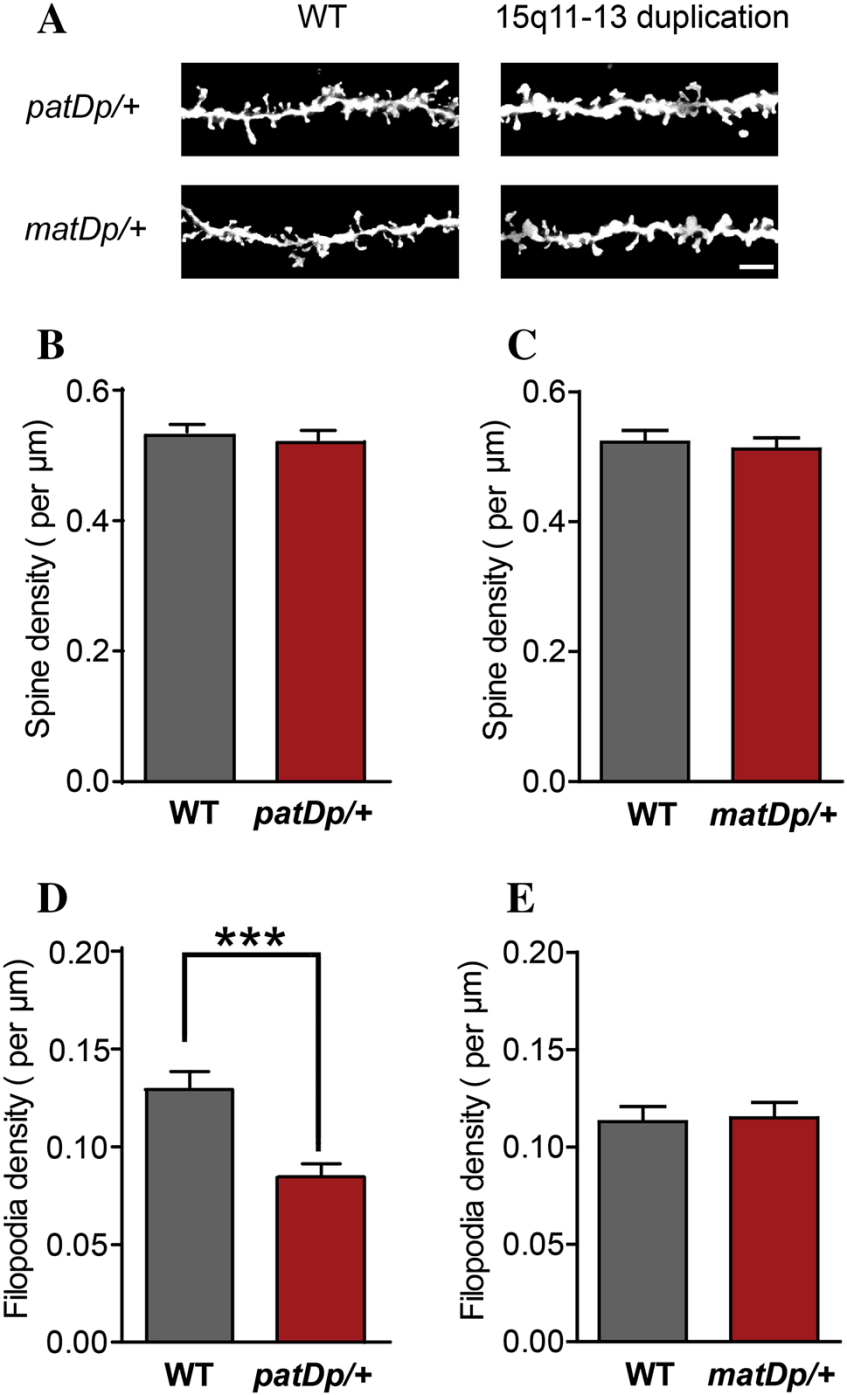
*patDp*/+ mice, but not *matDp*/+ mice, show impaired filapodia formation at P14. **A** Representative inverted Golgi staining images showing spines in basal dendrites of layer 2/3 pyramidal neurons in S1BF of P14 15q11-13 duplication mice, genotypes as indicated. *Scale bar* 5 μm. **B** Spine density in WT (*n* = 29) and *patDp*/+ (*n* = 32) mice at P14. **C** Spine density in WT (*n* = 25) and *matDp*/+ (*n* = 20) mice at P14. **D** Filopodia density in WT (*n* = 29) and *patDp*/+ (*n* = 32) mice at P14. **E** Filopodia density in WT (*n* = 25) and *matDp*/+ (*n* = 20) mice at P14. ***P < 0.001.

Is the spine pruning defect we observed in *MECP2* ^Tg1^ mice a common phenotype to multiple ASD models or specific to the *MECP2* duplication? To address this question and further explore spine pathology in ASD, we used another autism mouse model, in which the mouse chromosomal region corresponding to human chromosome 15q11-13 was engineered to be duplicated [38]. Since the duplicated region in these mice contains a series of imprinting genes expressed exclusively from the paternal or maternal copy (Fig. 3A) [36, 38], we separately examined spine density in mice carrying paternally (*patDp/+*) or maternally (*matDp/+*) inherited duplication. We found that at 1 month, spine density in *patDp/+* mice was significantly lower than that of wildtype littermates (*P* < 0.05; Fig. 3B, C) in S1BF, indicating impairment of spinogenesis in these *patDp/+* mice. Interestingly, *matDp/+* mice showed no significant differences in spine density as compared to wildtype littermates (*P* > 0.05; Fig. 3D, see also Discussion). To further characterize the defect in spinogenesis in *patDp/+* mice, we assayed spine density at the earlier age of P14 and found no significant differences (*P* > 0.05; Fig. 4A, B). At this age, spine density was also not affected in *matDp/+* mice (*P* > 0.05; Fig. 4A, C).

A considerable portion of spines come from filopodia that extend from the dendritic shaft and probe around for potential presynaptic partners. Once a filopodium “captures” a suitable axonal terminus, it could convert itself into a spine; otherwise, it likely retracts [17]. This transformation from filopodium to spine has been observed in cultured hippocampal neurons, as well as in brain slices, using live imaging [43, 44], and the synapse-like contacts made between axons and filopodia have been observed by electron microscopy [45]. Thus, dendritic filopodia contribute significantly to the formation of spines during early development of the brain, and the number of filopodia in early developmental stage may indicate the potential of a neuron to grow spines during spinogenesis. Since P14 is within the window of rapid filopodial dynamics, we also measured the density of filopodia (protrusions longer than 3 μm) in *patDp/+* mice, and found it to be significantly reduced (*P* < 0.001; Fig. 4D). This reduction likely contributes to the reduction in spine density at 1 month in these mice. Once again, no changes were observed in *matDp/+* mice (*P* > 0.05, Fig. 4E). Together, these results demonstrate a progressive impairment of spinogenesis that selectively occurs in mice with the paternally inherited 15q11-13 duplication.

### Spines are Less Mature in 15q11-13 Paternal Duplication Mice

**Fig. 5:**
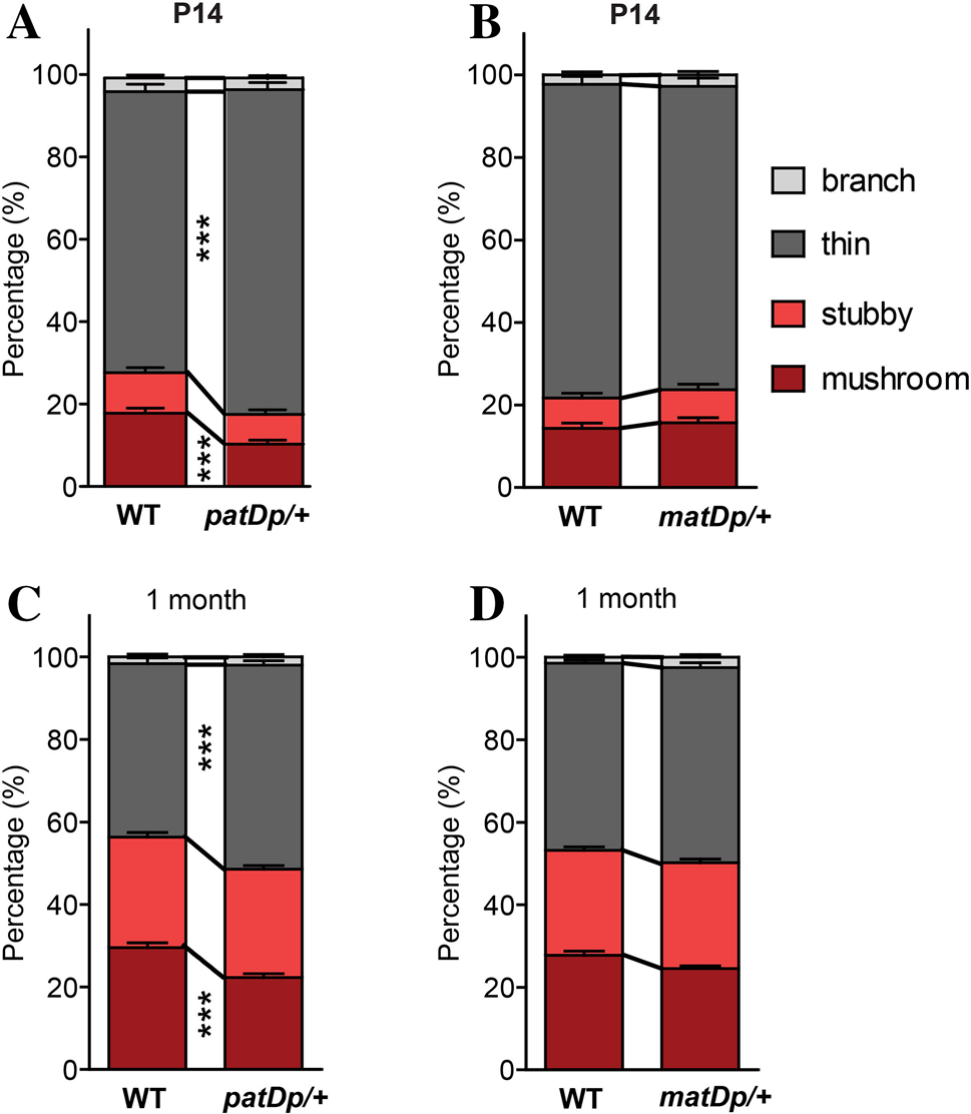
*patDp*/+ mice, but not matDp/+ mice, show impaired spinogenesis and delayed spine maturation. **A** Spine type classification in P14 WT and *patDp/*+ mice. **B** Spine type classification in P14 WT and *mat*Dp/+ mice. **C** Spine type classification in 1-month WT and *pat*Dp/+ mice. **D** Spine type classification in 1-month WT and *mat*Dp/+ mice. ***P < 0.001.

To examine spine maturation in 15q11-13 duplication mice, we analyzed spine morphology in *patDp/+* and *matDp/+* mice at P14 and 1 month. At P14, spines were mostly immature, as suggested by the large portion of thin spines (Fig. 5A, B). We found that in *patDp/+* mice, but not in *matDp/+* mice, the percentage of mushroom spines was reduced and that of thin spines increased correspondingly, as compared to wildtype littermates at P14 (Fig. 5A, B), despite similar spine density between the *patDp/+* mice and wildtype controls. At 1 month, the distribution pattern of spine subtypes was more mature than that at P14 in all genotypes (Fig. 5C, D vs. 5A, B). However, *patDp*/+ mice still possessed more thin spines and less mushroom spines than their wildtype littermates (Fig. 5C). Consistent with the spine density results, spine maturation was not affected in *matDp/+* mice at 1 month (Fig. 5D). Together, these results demonstrate that in addition to the impairment in generating spines, spines formed in *patDp*/+ mice were also less mature. The absence of these changes in *matDp/+* mice further underscores the selective impact of this chromosomal duplication depending on its genetic origin.

## Discussion

Gene dosage is critical to the normal development and functioning of the brain. This is particularly highlighted in ASD where both loss-of-function and overexpression of gene/chromosomal regions can lead to autistic phenotypes. Here we show that early spinogenesis and later spine pruning are respectively affected in two mouse models of autism with gene duplication, the paternally-inherited 15q11-13 duplication mice and the *MECP2* duplication mice. The distinct spine abnormalities in these two autism models likely reflect the diverse pathologies of the two types of ASDs, with the 15q11-13 duplication primarily impairing the initial establishment of neural connections and the *MECP2* duplication mainly targeting the later refinement of neural circuitry. Our results complement the findings of previous studies and provide further insight into our understanding of diverse pathologies in ASD at the level of synapses and neural circuits. These results raise the importance of time windows for the optimal therapeutic intervention in the treatment of distinct ASD subtypes. Spinogenesis and spine pruning at the population level in the sensory cortices could further serve as a model for assessing spine pathology in both animal models and postmortem ASD patient tissues, using simple morphological methods such as Golgi staining.

### Selective Abnormalities of Spines and Behaviors in 15q11-13 Paternal Duplication Mice

The duplication of chromosomal 15q11-13 region is one of the most frequently identified CNVs in ASD patients. It encompasses a series of imprinting genes including the maternally expressed *Ube3a* and *Atp10a*, the paternally expressed *Snrpn*, *Mkrn3*, *Magel2*, *Necdin* and *snoRNAs*, as well as non-imprinting genes such as a subset of GABA receptor subunits [36, 38] (Fig. 3A). Previous studies have shown that the *patDp/+* mice recapitulate some symptoms of human ASD patients, including impaired social interaction in the three-chamber test, behavioral inflexibility, decreased exploratory activity, impaired cerebellar plasticity and motor learning deficits [27, 36, 38, 46, 47]. Further investigation found that serotonin signaling decreased while dopamine signaling increased in these mice, indicating defects of the neuromodulation systems involved in emotion, motivation and social behaviors [36, 38, 48]. Consistent with the results of these functional analyses, we found that the *patDp/+* mice displayed significant and progressive defects in filopodia formation and spinogenesis (Figs. 3–5), which could lead to inadequate wiring of neural circuitry underlying the above-mentioned functions and behaviors. Consistently, a recent study using two-photon live imaging demonstrated increased spine turnover in *patDp/+* mice [49], suggesting that spines in these mice are more motile and unstable, which may account for the reduced spine density and immature spine morphology observed in these mice (Figs. 3, 5). It is somewhat surprising that all the behavioral, physiological and morphological abnormalities reported thus far [27, 36, 38, 39, 46–49], including ours (Figs. 3–5), were restricted to *patDp/+* mice, while the *matDp/+* mice seemed pretty normal, given that it is the maternal duplication of this chromosomal region that was thought to cause autism in humans [50]. Although a recent detailed human study showed that paternal duplications are also pathogenic and increase risks for ASD, developmental delay and multiple congenital anomalies [51], the “gap” between the mouse model and human patients is still a puzzling. We surmise that it may be due to the differences in imprinting status and epigenetic control of specific genes, in specific brain regions and between species [36]. In fact, some paternally expressed genes were found to be reduced in post-mortem brain tissues of individuals with maternal 15q11-q13 duplication [52], suggesting that gene expression within 15q11-q13 is not based entirely on copy number, and can be influenced by epigenetic mechanisms. Further research is required to elucidate the precise underlying mechanisms.

### Distinct Spine Abnormalities in Multiple ASD Mouse Models

ASD shows great diversity in both genetic etiology and clinical manifestation [5, 6, 8, 10, 26, 27]. How to bridge the gap between the genetic causes and the ASD symptoms poses a major challenge to autism research. Previous studies have demonstrated highly distinct spine phenotypes in multiple autism mouse models with gene deletion/mutation. For example, spine pruning defect was found in *Fragile X Mental Retardation 1* (*Fmr1*) knockout mice [53, 54], while decreased spine density was observed in *Mecp2* knockout mice throughout development [55–57]. Additionally, studies using neuronal cultures or transgenic mice have shown that spine density and/or shape were altered after genetic manipulation of proteins implicated in syndromic or non-syndromic autism, including neurexins/neuroligins, Shank2/3, Epac2, Tsc1/2, Ube3A and PTEN [6]. Here we further expand our knowledge of ASD spine pathology to two gene duplication mouse models. We note that our results demonstrating spine pruning defects in the basal dendrites of layer 2/3 neurons in the sensory cortices of *MECP2* ^Tg1^ mice (Figs. 1, 2) are consistent with and complementary to work by Jiang *et al*. in the apical dendrites of layer 5 neurons, where they showed a delayed pruning of spines on this dendritic segment in *MECP2* ^Tg1^ mice [58]. Thus, overproduction of Mecp2 likely results in a global effect across multiple layers of the cerebral cortex, to slow down or inhibit the spine pruning process during neural circuit refinement. We note that at 3 months, the pruning process was completely blocked in V1 (Fig. 2A, B) and only partially impaired in S1BF (Fig. 1A, B). Since mice reared under standard laboratory conditions likely use their tactile sensation more than their visual system, this result is consistent with the activity-dependence of spine pruning [14, 15].

Our existing knowledge of spine pathology in ASD suggests potentially two major classes of abnormalities: one is the insufficient genesis of spines seen in models including *Mecp2* knockout and 15q11-13 paternal duplication, which may result in a less connected and consequently inadequate neural circuitry; the other is defects in spine pruning as seen in *MeCP2* duplication and *Fmr1* knockout models, which may lead to an over-connected and thus less efficient neural circuitry in adulthood. We note that both the defects in spinogenesis and spine pruning, as we identified here, are developmentally regulated. The spine formation defect in 15q11-13 paternal duplication mice was not significant until 1 month, while the spinogenesis process seemed unaffected before the *MECP2* ^Tg1^ mice entered the spine pruning period. The progressive feature of these spine abnormalities is consistent with the gradually emerging and worsening of symptoms often seen in ASD patients. This diversity of spine abnormality in autism mouse models further raises the intriguing question of how distinct alterations of neural circuits during different developmental stages lead to common behavioral manifestations in ASD, including lack of social communications and repetitive behaviors.

### Insights from Another Gene Duplication Model

A good analogy of modeling gene-duplication-induced autism in mice is the mouse models of Down syndrome (DS), a disease caused by an extra copy of human chromosome 21 (Hsa21) and characterized by intellectual disability, deficits in learning and memory and early-onset Alzheimer’s disease [59–61]. Genes within the duplex region of Hsa21 are synthetic to 3 regions located on mouse chromosome 10 (Mmu10), Mmu16 and Mmu17 [59, 61]. Similar to DS patients, DS mouse models including Ts65Dn [62, 63] and Ts1Cje [64, 65], two most studied mouse models of DS, exhibit deficits in learning and hippocampal-dependent memory, impaired long-term potentiation (LTP) and altered excitatory/inhibitory balance [59, 61]. Interestingly, genes in Hsa21 have been shown to affect spine morphogenesis separately, and DS mouse models Ts65Dn, Ts1Cje and Ts1Rhr (trisomic region: Ts65Dn > Ts1Cje > Ts1Rhr) all show lowered spine density and enlarged spine head with gradually reduced severity (severity: Ts65Dn > Ts1Cje > Ts1Rhr) [59, 66], indicating an additive/synergic effect of these genes on spines. Given that decreased spine density is the shared pathological change in DS and in some ASD mouse models, including *Mecp2* knockout [55–57] and 15q11-13 duplication (Fig. 3), and that intellectual disability also occurs in a significant portion of ASD patients [5, 30, 66], it is interesting to consider the potential crosstalk between ASD genes and DS genes. In fact, it has been recently proposed that the product of one of the DS genes, DS critical region 1 (DSCR1), interacts with the Fragile X mental retardation protein (FMRP) to regulate the local protein synthesis in spines [66]. However, how ASD is linked to DS or other neuropsychiatric disorders mechanistically remains unclear and requires further investigation.

## References

[1] American Psychiatric Association. Diagnostic and statistical manual of mental disorders : DSM-5. Fifth edition. Washington, DC, United States of America, 2013.

[2] Ebert DH, Greenberg ME (2013). Activity-dependent neuronal signalling and autism spectrum disorder. Nature.

[3] Lee E, Lee J, Kim E. Excitation/inhibition imbalance in animal models of autism spectrum disorders. Biol Psychiatry 2016. 10.1016/j.biopsych.2016.05.011 27450033

[4] Mullins C, Fishell G, Tsien RW (2016). Unifying views of autism spectrum disorders: a consideration of autoregulatory feedback loops. Neuron.

[5] Zoghbi HY, Bear MF. Synaptic dysfunction in neurodevelopmental disorders associated with autism and intellectual disabilities. Cold Spring Harb Perspect Biol 2012, 4. 10.1101/cshperspect.a009886 PMC3282414 22258914

[6] Penzes P, Cahill ME, Jones KA, VanLeeuwen JE, Woolfrey KM (2011). Dendritic spine pathology in neuropsychiatric disorders. Nat Neurosci.

[7] Nelson SB, Valakh V (2015). Excitatory/inhibitory balance and circuit homeostasis in autism spectrum disorders. Neuron.

[8] de la Torre-Ubieta L, Won H, Stein JL, Geschwind DH (2016). Advancing the understanding of autism disease mechanisms through genetics. Nat Med.

[9] Geschwind DH, State MW (2015). Gene hunting in autism spectrum disorder: on the path to precision medicine. Lancet Neurol.

[10] Sztainberg Y, Zoghbi HY (2016). Lessons learned from studying syndromic autism spectrum disorders. Nat Neurosci.

[11] Gray EG (1959). Electron microscopy of synaptic contacts on dendrite spines of the cerebral cortex. Nature.

[12] Rakic P, Bourgeois JP, Eckenhoff MF, Zecevic N, Goldman-Rakic PS (1986). Concurrent overproduction of synapses in diverse regions of the primate cerebral cortex. Science.

[13] Rakic P, Bourgeois JP, Goldman-Rakic PS (1994). Synaptic development of the cerebral cortex: implications for learning, memory, and mental illness. Prog Brain Res.

[14] Bhatt DH, Zhang S, Gan WB (2009). Dendritic spine dynamics. Annu Rev Physiol.

[15] Bian WJ, Miao WY, He SJ, Qiu Z, Yu X (2015). Coordinated spine pruning and maturation mediated by inter-spine competition for cadherin/catenin complexes. Cell.

[16] Elston GN, Oga T, Fujita I (2009). Spinogenesis and pruning scales across functional hierarchies. J Neurosci.

[17] Yuste R, Bonhoeffer T (2004). Genesis of dendritic spines: insights from ultrastructural and imaging studies. Nat Rev Neurosci.

[18] Zuo Y, Lin A, Chang P, Gan WB (2005). Development of long-term dendritic spine stability in diverse regions of cerebral cortex. Neuron.

[19] Huttenlocher PR (2002). Neural plasticity: the effects of environment on the development of the cerebral cortex.

[20] Grutzendler J, Kasthuri N, Gan WB (2002). Long-term dendritic spine stability in the adult cortex. Nature.

[21] Holtmaat AJ, Trachtenberg JT, Wilbrecht L, Shepherd GM, Zhang X, Knott GW (2005). Transient and persistent dendritic spines in the neocortex in vivo. Neuron.

[22] Harris KM, Jensen FE, Tsao B (1992). Three-dimensional structure of dendritic spines and synapses in rat hippocampus (CA1) at postnatal day 15 and adult ages: implications for the maturation of synaptic physiology and long-term potentiation. J Neurosci.

[23] Harris KM, Weinberg RJ (2012). Ultrastructure of synapses in the mammalian brain. Cold Spring Harb Perspect Biol.

[24] Tonnesen J, Katona G, Rozsa B, Nagerl UV (2014). Spine neck plasticity regulates compartmentalization of synapses. Nat Neurosci.

[25] Yuste R (2013). Electrical compartmentalization in dendritic spines. Annu Rev Neurosci.

[26] Devlin B, Scherer SW (2012). Genetic architecture in autism spectrum disorder. Curr Opin Genet Dev.

[27] Huguet G, Ey E, Bourgeron T (2013). The genetic landscapes of autism spectrum disorders. Annu Rev Genomics Hum Genet.

[28] Krumm N, O’Roak BJ, Shendure J, Eichler EE (2014). A de novo convergence of autism genetics and molecular neuroscience. Trends Neurosci.

[29] Nakai N, Otsuka S, Myung J, Takumi T (2015). Autism spectrum disorder model mice: Focus on copy number variation and epigenetics. Sci China Life Sci.

[30] Coe BP, Girirajan S, Eichler EE (2012). The genetic variability and commonality of neurodevelopmental disease. Am J Med Genet C Semin Med Genet.

[31] Amir RE, Van den Veyver IB, Wan M, Tran CQ, Francke U, Zoghbi HY (1999). Rett syndrome is caused by mutations in X-linked MECP2, encoding methyl-CpG-binding protein 2. Nat Genet.

[32] Chahrour M, Zoghbi HY (2007). The story of Rett syndrome: from clinic to neurobiology. Neuron.

[33] Lombardi LM, Baker SA, Zoghbi HY (2015). MECP2 disorders: from the clinic to mice and back. J Clin Investig.

[34] Ramocki MB, Peters SU, Tavyev YJ, Zhang F, Carvalho CM, Schaaf CP (2009). Autism and other neuropsychiatric symptoms are prevalent in individuals with MeCP2 duplication syndrome. Ann Neurol.

[35] Ramocki MB, Tavyev YJ, Peters SU (2010). The MECP2 duplication syndrome. Am J Med Genet A.

[36] Takumi T (2011). The neurobiology of mouse models syntenic to human chromosome 15q. J Neurodev Disord.

[37] Collins AL, Levenson JM, Vilaythong AP, Richman R, Armstrong DL, Noebels JL (2004). Mild overexpression of MeCP2 causes a progressive neurological disorder in mice. Hum Mol Genet.

[38] Nakatani J, Tamada K, Hatanaka F, Ise S, Ohta H, Inoue K (2009). Abnormal behavior in a chromosome-engineered mouse model for human 15q11-13 duplication seen in autism. Cell.

[39] Amir RE, Van den Veyver IB, Wan M, Tran CQ, Francke U, Zoghbi HY (1999). Rett syndrome is caused by mutations in X-linked MECP2, encoding methyl-CpG-binding protein 2. Nat Genet.

[40] Samaco RC, Mandel-Brehm C, McGraw CM, Shaw CA, McGill BE, Zoghbi HY (2012). Crh and Oprm1 mediate anxiety-related behavior and social approach in a mouse model of MECP2 duplication syndrome. Nat Genet.

[41] Sztainberg Y, Chen HM, Swann JW, Hao S, Tang B, Wu Z (2015). Reversal of phenotypes in MECP2 duplication mice using genetic rescue or antisense oligonucleotides. Nature.

[42] Liu Z, Li X, Zhang JT, Cai YJ, Cheng TL, Cheng C (2016). Autism-like behaviours and germline transmission in transgenic monkeys overexpressing MeCP2. Nature.

[43] Dailey ME, Smith SJ (1996). The dynamics of dendritic structure in developing hippocampal slices. J Neurosci.

[44] Ziv NE, Smith SJ (1996). Evidence for a role of dendritic filopodia in synaptogenesis and spine formation. Neuron.

[45] Fiala JC, Feinberg M, Popov V, Harris KM (1998). Synaptogenesis via dendritic filopodia in developing hippocampal area CA1. J Neurosci.

[46] Tamada K, Tomonaga S, Hatanaka F, Nakai N, Takao K, Miyakawa T (2010). Decreased exploratory activity in a mouse model of 15q duplication syndrome; implications for disturbance of serotonin signaling. PLoS One.

[47] Piochon C, Kloth AD, Grasselli G, Titley HK, Nakayama H, Hashimoto K (2014). Cerebellar plasticity and motor learning deficits in a copy-number variation mouse model of autism. Nat Commun.

[48] Farook MF, DeCuypere M, Hyland K, Takumi T, LeDoux MS, Reiter LT (2012). Altered serotonin, dopamine and norepinepherine levels in 15q duplication and Angelman syndrome mouse models. PLoS One.

[49] Isshiki M, Tanaka S, Kuriu T, Tabuchi K, Takumi T, Okabe S (2014). Enhanced synapse remodelling as a common phenotype in mouse models of autism. Nat Commun.

[50] Cook EH, Lindgren V, Leventhal BL, Courchesne R, Lincoln A, Shulman C (1997). Autism or atypical autism in maternally but not paternally derived proximal 15q duplication. Am J Hum Genet.

[51] Isles AR, Ingason A, Lowther C, Walters J, Gawlick M, Stober G (2016). Parental origin of interstitial duplications at 15q11.2-q13.3 in schizophrenia and neurodevelopmental disorders. PLoS Genet.

[52] Scoles HA, Urraca N, Chadwick SW, Reiter LT, Lasalle JM (2011). Increased copy number for methylated maternal 15q duplications leads to changes in gene and protein expression in human cortical samples. Mol Autism.

[53] Comery TA, Harris JB, Willems PJ, Oostra BA, Irwin SA, Weiler IJ (1997). Abnormal dendritic spines in fragile X knockout mice: maturation and pruning deficits. Proc Natl Acad Sci U S A.

[54] Galvez R, Greenough WT (2005). Sequence of abnormal dendritic spine development in primary somatosensory cortex of a mouse model of the fragile X mental retardation syndrome. Am J Med Genet A.

[55] Xu X, Miller EC, Pozzo-Miller L (2014). Dendritic spine dysgenesis in Rett syndrome. Front Neuroanat.

[56] Stuss DP, Boyd JD, Levin DB, Delaney KR (2012). MeCP2 mutation results in compartment-specific reductions in dendritic branching and spine density in layer 5 motor cortical neurons of YFP-H mice. PLoS One.

[57] Chao HT, Zoghbi HY, Rosenmund C (2007). MeCP2 controls excitatory synaptic strength by regulating glutamatergic synapse number. Neuron.

[58] Jiang M, Ash RT, Baker SA, Suter B, Ferguson A, Park J (2013). Dendritic arborization and spine dynamics are abnormal in the mouse model of MECP2 duplication syndrome. J Neurosci.

[59] Ruparelia A, Pearn ML, Mobley WC (2013). Aging and intellectual disability: insights from mouse models of Down syndrome. Dev Disabil Res Rev.

[60] Antonarakis SE, Lyle R, Dermitzakis ET, Reymond A, Deutsch S (2004). Chromosome 21 and down syndrome: from genomics to pathophysiology. Nat Rev Genet.

[61] Liu C, Belichenko PV, Zhang L, Fu D, Kleschevnikov AM, Baldini A (2011). Mouse models for Down syndrome-associated developmental cognitive disabilities. Dev Neurosci.

[62] Davisson MT, Schmidt C, Akeson EC (1990). Segmental trisomy of murine chromosome 16: a new model system for studying Down syndrome. Prog Clin Biol Res.

[63] Reeves RH, Irving NG, Moran TH, Wohn A, Kitt C, Sisodia SS (1995). A mouse model for Down syndrome exhibits learning and behaviour deficits. Nat Genet.

[64] Sago H, Carlson EJ, Smith DJ, Kilbridge J, Rubin EM, Mobley WC (1998). Ts1Cje, a partial trisomy 16 mouse model for Down syndrome, exhibits learning and behavioral abnormalities. Proc Natl Acad Sci USA.

[65] Sago H, Carlson EJ, Smith DJ, Rubin EM, Crnic LS, Huang TT (2000). Genetic dissection of region associated with behavioral abnormalities in mouse models for Down syndrome. Pediatr Res.

[66] Chang KT, Ro H, Wang W, Min KT (2013). Meeting at the crossroads: common mechanisms in Fragile X and Down syndrome. Trends Neurosci.

